# Digital Image Correlation of 2D X-ray Powder Diffraction Data for Lattice Strain Evaluation

**DOI:** 10.3390/ma11030427

**Published:** 2018-03-15

**Authors:** Hongjia Zhang, Tan Sui, Enrico Salvati, Dominik Daisenberger, Alexander J. G. Lunt, Kai Soon Fong, Xu Song, Alexander M. Korsunsky

**Affiliations:** 1 Department of Engineering Science, University of Oxford, Oxford OX1 3PJ, UK; hongjia.zhang@eng.ox.ac.uk (H.Z.); enrico.salvati@eng.ox.ac.uk (E.S.); 2 Department of Mechanical Engineering Sciences, University of Surrey, Guildford GU2 7XH, UK; tan.sui@eng.ox.ac.uk; 3 Beamline I15, Diamond Light Source, Didcot OX11 0DE, UK; dominik.daisenberger@diamond.ac.uk; 4 CERN, CH-1211 Geneva 23, Switzerland; alexander.lunt@cern.ch; 5 Singapore Institute of Manufacturing Technology (SIMTech), 73 Nanyang Drive, Singapore 637662, Singapore; ksfong@simtech.a-star.edu.sg (K.S.F.); xsong@simtech.a-star.edu.sg (X.S.)

**Keywords:** 2D X-ray powder diffraction, strain measurement, Digital Image Correlation, 3-point bending

## Abstract

High energy 2D X-ray powder diffraction experiments are widely used for lattice strain measurement. The 2D to 1D conversion of diffraction patterns is a necessary step used to prepare the data for full pattern refinement, but is inefficient when only peak centre position information is required for lattice strain evaluation. The multi-step conversion process is likely to lead to increased errors associated with the ‘caking’ (radial binning) or fitting procedures. A new method is proposed here that relies on direct Digital Image Correlation analysis of 2D X-ray powder diffraction patterns (XRD-DIC, for short). As an example of using XRD-DIC, residual strain values along the central line in a Mg AZ31B alloy bar after 3-point bending are calculated by using both XRD-DIC and the conventional ‘caking’ with fitting procedures. Comparison of the results for strain values in different azimuthal angles demonstrates excellent agreement between the two methods. The principal strains and directions are calculated using multiple direction strain data, leading to full in-plane strain evaluation. It is therefore concluded that XRD-DIC provides a reliable and robust method for strain evaluation from 2D powder diffraction data. The XRD-DIC approach simplifies the analysis process by skipping 2D to 1D conversion, and opens new possibilities for robust 2D powder diffraction data analysis for full in-plane strain evaluation.

## 1. Introduction

X-ray powder diffraction (XRD) is a widely used experimental technique that finds extensive application in materials engineering [1,2,3,4,5,6,7,8,9,10]. XRD can be used for the quantification of phase composition, preferred grain orientation (texture), and also offers a powerful non-destructive way to measure lattice strain in a variety of crystalline materials [11,12,13,14]. Residual stresses that remain in a material after various processing operations have a significant influence on the mechanical properties and structural performance of materials and components. The relationship between residual stresses and lattice strains has been the subject of extensive study over the last century [15,16], and led to the appreciation of the multi-scale nature of these phenomena [17], and the refinement of modelling tools that assist the conversion from XRD measurements to strain evaluation and residual stress calculation. The presence of residual strain changes the interplanar lattice spacing with respect to the strain-free state, and affects the XRD pattern by causing a systematic shift of the peak centre, so that careful analysis of diffraction pattern changes can be used to quantify lattice strain [18].

One advantage of XRD is its non-destructive nature, which means no material removal is involved in the experiment, unlike other destructive or semi-destructive methods such as hole-drilling [19], FIB-DIC ring-core milling [20,21], slitting [22] or layer removal [23], etc. XRD is also eminently suitable for in situ strain measurement that can be done to observe and quantify the strain evolution during thermal and deformation processing. An additional advantage of the high energy synchrotron XRD method is that it can penetrate the sample and provide information about the average bulk strain, as opposed to other methods such as strain gauge analysis [24] or laboratory X-ray diffraction [25] which can only evaluate strains at sample surface.

It is convenient to distinguish between synchrotron-based and conventional laboratory XRD, due to the vast difference in flux and resolution that can be typically attained using these approaches. Although lab-based X-ray equipment is more commonly available, it has significant limitations in terms of sample thickness, spatial resolution and data collection time. High energy synchrotron X-ray transmission configuration relies on high flux, brightness, energy and penetration ability, making it possible to use micro-scale beam sizes and achieve better spatial resolution [26]. In the present study we leave aside the single crystal Laue XRD configuration [27], and concentrate on polycrystalline XRD. The interaction between an incident collimated monochromatic X-ray beam and a polycrystalline aggregate or powder leads to a series of scattering cones that produce concentric Debye-Scherrer rings on a 2D detector mounted coaxially with the beam. Obtaining precise strain values requires careful interpretation of 2D XRD data so that the radial peak position is determined with the accuracy approaching $10^{-4}$ or better. Developing reliable and efficient methods for 2D XRD pattern interpretation is one of the key remaining challenges in the area of XRD strain analysis.

Conventional data analysis is a multi-stage process which involves three main steps: calibration, conversion of the 2D pattern(s) into a 1D profile(s), and Gaussian fitting for peak centre determination to calculate strain values.

In the first step, calibration is performed for sample-detector distance, image centre of diffraction pattern as well as corrections for the geometrical distortion due to detector’s orientation. Errors are inevitable during calibration and interpretation. They can arise when collecting diffraction pattern from the positioning of a standard calibration sample, or in the subsequent calculation that involves data reduction and manipulation. This is the first error source. Microstrains normally concern sub-pixel ring movement. Therefore, it is very likely that when the magnitude of micro-level residual strain itself is not large enough, errors become dominant and cover up the real value of residual strain.

For the second step, 2D diffraction patterns are converted into 1D intensity-2θ profiles. By putting upper and lower limits on the angle range of azimuthal integration a sector (similar to a ‘cake slice’) for integration is defined. The equivalent 1D profile extracted from this sector is the strain towards the direction at the average angle of upper and lower limits. The way to obtain a strain along a specific angle is often informally referred to as ‘caking’ [28]. Nevertheless, the ‘caking’ approach is not entirely straightforward, as it requires defining sectors for azimuthal integration range, selecting directions, etc. On top of this, the results from this analysis are based on an average of the whole section which is imprecise in the case of textured sample (whose ring intensity is not uniform). This is the second error source.

Step three is to calculate strain values from tracking peak positions on 1D intensity-2θ plot. Peak positions are attained by Gaussian fitting of the integrated profiles. Note that peak centre obtained here is in 2θ value. *d*-spacing, the distance between adjacent lattice planes, is related to 2θ according to Bragg’s law

(1) 2dsinθ=nλ

where λ is the wavelength of the incident X-ray and n is a positive integer. The relationship between residual strain and d-spacing is given by

(2) $$\epsilon =\left(d-d_{0}\right)/d_{0}$$

where ϵ is elastic residual strain, *d* is *d*-spacing of the processed sample and $d_{0}$ is the value of *d*-spacing when the sample is strain free [29]. Nonetheless, useful Gaussian peak fitting demands continuity and symmetry of peaks, which require high quality Debye-Scherrer rings with high intensity and good uniformity. However, this is not always possible due to the nature of texture introduced in the processed sample or the lack of sufficient grain sampling by the beam (within the gauge volume). As a consequence, the 1D plot converted from grainy diffraction pattern has poor quality peaks and this leads to inaccurate or even unsuccessful Gaussian peak fitting. This is the third error source.

For convenience, the 3-step method described above is referred to as ConFit (conversion + Gaussian fitting) method in the following text. The determination of the absolute peak position involves errors from the three sources discussed above. The limitations of ConFit method result from the fact that conversion from the 2D diffraction pattern to 1D plot followed by fitting is required. On the other hand, the interpretation of each 1D plot provides complete information including peak position, peak width, peak shape and relative intensity. However, complete information is only sought in the more complicated full pattern analysis which seeks to evaluate phase composition, texture, grain size and strains. In contrast, strain measurement only relies on the relative change in the lattice parameter that corresponds to the radial relative position shift of the ring segment in the diffraction pattern. Therefore, tracking the ring shift only is sufficient for strain evaluation, and may be accomplished avoiding the 2D to 1D conversion.

Digital Image Correlation (DIC) offers a means of determining the relative displacement between two or more 2D images. In recent years, DIC has been newly adopted for the treatment of Laue diffraction data. Petita et al. [30] presented Laue-DIC as a new method which is able to determine the Laue spot motion field and obtain the local elastic strain increment and the relative lattice rotation. Later on, enhanced Laue-DIC was developed by Zhang et al. [31]. The improvement of enhanced Laue-DIC is mainly reflected on two aspects: the capability of calculating (i) the deviatoric elastic strain and orientation of the lattice (ii) the calibration parameters. Nonetheless, to the best of our knowledge, so far no work has been reported that utilises DIC to analyse 2D XRD patterns for strain evaluation. In this paper XRD-DIC is proposed as a method of analysing 2D XRD data for full in-plane strain measurement. XRD-DIC utilises DIC technique to analyse 2D XRD patterns directly, without 2D to 1D conversion, and has the advantages of being robust, easy to use, and accurate. As an illustration, XRD-DIC is used here to analyse high energy synchrotron 2D XRD data attained from the central line scan in a magnesium alloy AZ31B bar after 3-point bending.

## 2. The XRD-DIC Approach

### 2.1. DIC Introduction

DIC provides non-contact measurement of the displacement field on sample surfaces by using image processing methods and numerical computing to compare sequential images [32]. DIC was originally developed for tracking particles moving in flow (velocimetry). With the development of imaging techniques and increased processing power in computers it became possible to improve DIC resolution to allow the detection of the small displacements associated with both elastic and plastic deformation of solids. There is currently a broad range of applications of DIC in science and engineering for measuring displacements and evaluating strains at both the microscopic and macroscopic level. The application of DIC in combination with FIB ring-core milling allows the evaluation of residual strain at the microscopic scale [20,21,33]. In macroscopic mechanical experiments, such as crack opening experiments [34,35], DIC is used to locate cracks and perform precise crack opening measurements [36]. Moreover, DIC can be utilised to monitor displacements of large engineering structures [37].

### 2.2. DIC Implementation

DIC determines the displacement field of the region of interest (ROI) in a deformed image by comparing it with the reference image. A variety of DIC packages are available, such as Opencv, Ncorr and DaVis. The DIC package used herein is a Matlab compiled version developed by C. Eberl and M. Senn [38] which includes pre-processing, tracking and post-processing of the data.

#### 2.2.1. Pre-Processing

**Figure 1:**
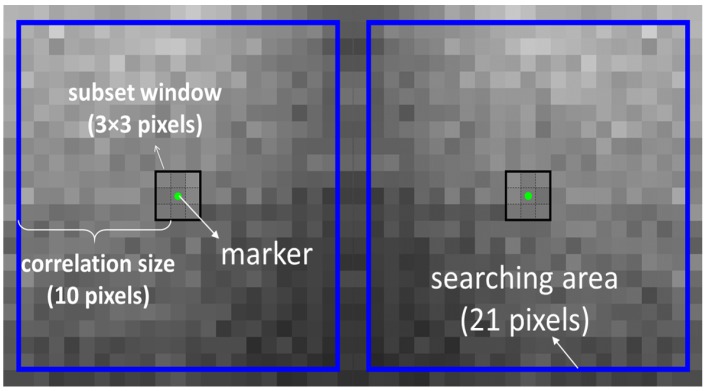
Schematic demonstration of definitions of subset window, marker, correlation size and searching area in the DIC performed.

In this compiled version, a sequence of images can be saved into a file named ‘filelist’ for the purpose of analysing multiple images in one DIC analysis. The first image in the image sequence is called the base image, which acts as the reference image. The arrangement and the position of markers (e.g., evenly distributed in a rectangle area or on a line) can be defined by choosing ROI on the base image, and then the position of each marker are written in files ‘gridx’ and ‘gridy’ with x and y coordinates (unit: pixel) as its initial position. As indicated in Figure 1 two markers (green dots) are placed. Each marker occupies one pixel and is associated with a particular subset window. In the current analysis subset window was selected to be a square area with size 3 × 3 pixels.

Aside from placing markers, a parameter ’Correlation Size’ (*corrsize*) needs to be set for DIC. It has a unit of pixel and limits the size of ‘searching area’ (blue squares in Figure 1). Searching area is the square region around a marker within which the marker is being tracked. The size of searching area is 2 × *corrsize* + 1, where 1 pixel comes from the pixel occupied by the marker. In Figure 1, *corrsize* is 10 pixels and subset window size is 21 pixels.

#### 2.2.2. Tracking and Post-Processing

In the tracking process, subsequent images are sequentially compared with the base image, in order to determine the displacement field of the deformed ROI in each image. Displacement field of ROI is described with the displacement of each marker distributed in the ROI. Note that each marker corresponds to its own 3 × 3 subset window. By moving the subset window of a marker around its searching area, the cross-correlation surface can be calculated. The details of cross-correlation surface calculation are explained in the literature [39]. The new position of the marker is found as the peak position of the cross-correlation surface. Integer pixel accuracy can be determined by simply locating the coordinates which have the maximum value in the cross correlation surface, whilst sub-pixel accuracy is achieved by peak fitting of the cross-correlation surface. In practice the local shape of the correlation function landscape in the vicinity of the peak can be sufficiently well approximated by a low (e.g., second) order polynomial. This facilitates the determination of the peak position to a resolution typically better than 0.05 pixel [39]. The quality of the peak is expressed by the correlation coefficient (*cc*) which is equal to the maximum magnitude of the cross-correlation function whose maximum value is 1. Another important measure of the marker tracking effectiveness is the standard deviation (*sd*) of peak position which shows the displacement precision. Images with good contrast and low noise lead to sharp peaks with *cc* magnitude close to 1 and small peak width (hence a low *sd*) in the cross-correlation surface. This leads to low error in the evaluation of the relative displacement between images. The new positions of each marker on a series of deformed images are recorded and the relative displacement (both in the x- and y- directions) is obtained by comparing it to the initial positions in the base image.

If a marker has a relatively large *sd*, small (near zero) *cc* or large displacement with respect to its neighbours, it means its quality of tracking is poor. For this reason, marker ‘cleaning’ is performed to eliminate those markers which exceed particular thresholds. It turned out that in this work only 13 out of 279 markers (4.66%) were removed (“cleaned”) due to ineffective DIC tracking. This also indicates that the DIC results have good robustness.

The application of DIC on 2D XRD patterns (XRD-DIC) is outlined in detail below by way of an example: evaluating residual strains of the central line in a 3-point-bent bar.

### 2.3. Advantages of XRD-DIC

The major advantage of using DIC to analyse 2D X-ray powder diffraction patterns (Debye-Scherrer rings) is that it can avoid the necessity of 2D to 1D conversion and fitting. This means that errors due to calibration, conversion and fitting can be minimised. Moreover, the effort-intensive radial-azimuthal binning exercise often referred to as ‘caking’ is no longer needed to attain strains in different directions. Instead, in the XRD-DIC approach, the lattice strains in an azimuthal direction of interest can be evaluated by tracking the movement of the ring segment in this direction, extracting the radial component of displacement, and dividing it by the original ring radius to quantify lattice strain. This approach is easier and more straightforward, since ‘caking’ typically performs binning and averaging within an entire sector (‘slice’ of the ‘cake’), whilst XRD-DIC only seeks to interpret information about the immediate vicinity of the selected markers, and is therefore more convenient and even potentially more precise in this sense. Furthermore, full in-plane strain analysis can be readily accomplished [40] in one DIC analysis by selecting as many ring segments as necessary and calculating strains, from which principal directions and principal strains can be determined. In principle, XRD-DIC is likely to have the capability to measure lattice strains at the accuracy that can reach ∼$10^{-5}$.

In this paper the use of DIC for 2D XRD data strain analysis is proposed, implemented and discussed. The experimental example chosen to illustrate strain evaluation by XRD-DIC is an X-ray line scan along the central line in a 3-point bending experiment. The material selected was magnesium alloy AZ31B which had previously processed using Constrained Groove Pressing (CGP, a flavour of Severe Plastic Deformation, SPD [41]) in order to promote grain refinement.

## 3. Material and Experiment

### 3.1. Three-Point Bending

**Figure 2:**
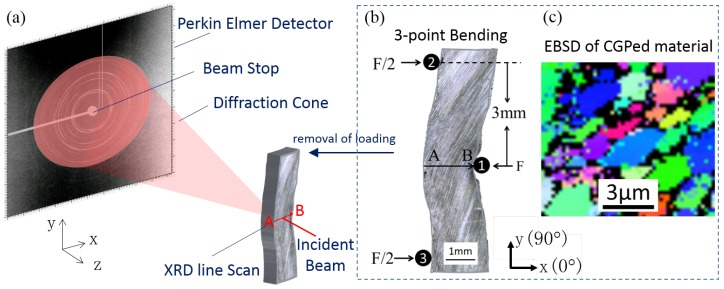
(**a**) Layout of XRD experiment; (**b**) Set-up of 3-point bending and (**c**) EBSD of as-CGPed sample.

Mg AZ31B alloy is important for use in ‘lightweighting’ in electronics and automotive applications. One of the challenges for the wider use of these alloys is its inherently large grain size that leads to relatively poor mechanical properties. Grain size can be refined either by alloying [42] or by SPD [43]. CGP, as a method of SPD, was used to prepare samples and fine grains of the size in the range of ∼2–5 μm were obtained. The microstructure is illustrated in Figure 2c in the form of an EBSD (electron backscatter diffraction) map of the sample cross-section after CGP.

A 1.8 mm wide and 2.1 mm thick bar cut from a CGP-processed magnesium AZ31B alloy plate was deformed by 3-point bending using a 5kN Deben (MT10223, 5 kN Extended Tensile Tester), as illustrated in in Figure 2b. The distance between the pins was 3 and 6 mm, respectively, and a force of 400 N was applied to ensure sufficient plastic deformation was introduced in the sample. After removal of bending load, the sample was subjected to X-ray diffraction scanning at Beamline I15, Diamond Light Source, UK.

### 3.2. XRD Experiment

**Figure 3:**
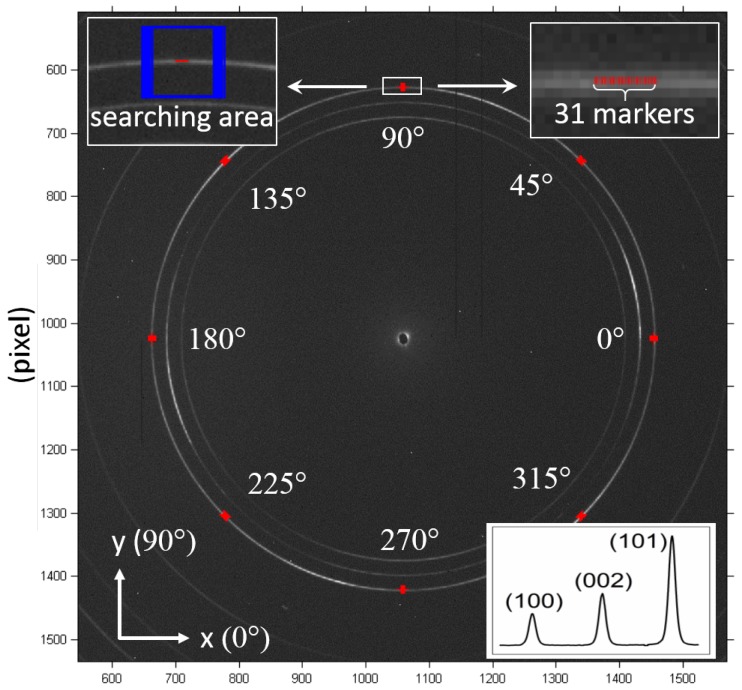
DIC setup illustrated for the base image with markers placed at different azimuthal angles on the (101) diffraction ring. The markers and searching area for the $90^{\circ }$ azimuthal angle are shown at a higher magnification in the figure inserts as an example.

The layout of the XRD experiment is illustrated in Figure 2a. The beam size of X-ray used was 20 μm × 20 μm to ensure that there were a sufficient number of grains within the gauge volume to produce enough powder averaging and thereby good statistics. The resulting Debye-Scherrer rings were mostly continuous, which ensured that after the conversion of 2D data to 1D plots, the bell-shaped diffraction peaks have sufficient resolution to facilitate high precision Gaussian fitting. The inset on the bottom right of Figure 3 demonstrates the equivalent 1D plot obtained from the base image and serves to illustrate the peak shapes obtained. A monochromatic beam with an energy of 72 keV and cross sectional size of 20μm×20μm was scanned along the central line of 3-point bending sample between points A and B as shown in Figure 2b. The step size of the line scan was 10μm, and 180 data points were collected (a sequence of 180 diffraction patterns was acquired), covering the entire width of the bar. The beam size was twice the step size and therefore there was 50% overlap between successive steps. A Perkin-Elmer flat panel detector 1621 EN (Wiesbaden, Germany) with 0.2 mm × 0.2 mm pixel size was placed 1125.5 mm away from the sample for 2D diffraction data collection. The matrix of the detector used in this study was 2048 × 2048 pixels. With the smallest reliably detectable ring shift of 0.05 (typical for sub-pixel accuracy DIC) and the image size of 2048 pixels, the accuracy of lattice strains can reach $∼10^{-5}$.

### 3.3. DIC Analysis

In order to perform reliable DIC analysis image processing needs to be applied to the images before automated tracking is performed. This process is critical as the DIC process is reliant upon comparing the intensity of different images, and therefore it is important to maintain image intensity throughout the scan. In this study it was evident that electronic noise (dark field) of the detector had an impact on the results obtained and hence needed to be considered during the image processing stages. A single dark field image was subtracted from each pattern of the XRD line scan in order to correct variations in detector sensitivity. Furthermore, image filtering was performed using ‘2D Gaussian’ and ‘Contrast’ filters. Both these filters are available in the MathWorks Image Processing Toolbox [44] and are embedded in the DIC code used [38]. The purpose of this automatic step in the DIC algorithm is to optimise image quality for subsequent DIC analysis. Image pre-processing was evaluated by monitoring the presence of sharp peak with small *sd* in the cross-correlation surface for a better confidence in fitting accuracy.

DIC was performed on the 3rd ring of the diffraction pattern that corresponds to crystallographic plane normal with the Miller index (1 0 1). The first image of the sequence of 180 diffraction patterns was used as the base image from which all marker displacements were calculated. The coordinates of the markers were calculated using the ring radius and azimuthal angle (β) with respect to the image centre coordinates, and saved into ‘gridx’ and ‘gridy’ data files. Figure 3 illustrates the positioning of the markers on the base image. Eight ring segments were chosen, corresponding to the azimuthal angles $0^{\circ }$, $45^{\circ }$, $90^{\circ }$, $135^{\circ }$, $180^{\circ }$, $225^{\circ }$, $270^{\circ }$ and $315^{\circ }$. For each ring segment, 31 markers were uniformly placed within the central angle range of 1°. A higher magnification image of the markers positioned at an azimuthal angle 90° are shown in the top right inset of Figure 3 as an example. The radial shift of each of the marker positions (for the 8 ring segments) was determined using DIC, and then converted to strains based on Equations (1) and (2) as described in more detail below. This complete range of strain values served as input for the computation of the full in-plane strain tensor. Note that the coordinate system related to both the sample and laboratory coordinates was used throughout the analysis (Figure 2a).

In the DIC analysis performed, the *corrsize* parameter value was set to 20 pixels which corresponds to a square searching area of size 41 × 41 pixels. The principle of choosing *corrsize* for XRD-DIC analysis is to ensure that each marker could be tracked in a large enough area, while avoiding aliasing (false matches) that arise from other high intensity features (rings) within the searching area. The searching area for the markers located at an azimuthal angle of $90^{\circ }$ is illustrated in Figure 3, which can be seen to cover all the space between the 2nd ring and the 3rd ring while excluding the 2nd ring.

The output of the XRD-DIC analysis is the new matched shifted coordinates of each marker. By comparing a marker’s new position with its initial position, the radial displacement can be calculated. The radial displacement of the 3rd ring on each image, $\bar{dp}$, is calculated by averaging the radial displacement of markers:

(3) $$\bar{dp}=\frac{1}{n}\Sigma_{i=1}^{n}\sqrt{{\left(p_{i}^{\prime }-p_{i}\right)}^{2}}$$

where $p_{i}^{\prime }$ and $p_{i}$ denote the marker radial position in the current and base images, respectively.

The equation for elastic lattice strain calculation can be obtained from Equations (1) and (2), which is shown as follows:

(4) $$\epsilon =-\frac{1}{2}\frac{{\left(\frac{p_{0}}{D}\right)}^{2}}{\sqrt{1+{\left(\frac{p_{0}}{D}\right)}^{2}}-1}\cos^{2}\left(2\theta \right)\frac{dp}{p_{0}}\approx -\frac{dp}{p_{0}}$$

where $p_{0}$ (mm) is the radial distance between the ring and the image centre in the strain-free condition (similar to the role of $d_{0}$ in Equation (2)), *D* (mm) is the sample-detector distance and 2θ is the angle between incident and diffracted X-rays. Note that the pixel area is equal to 0.2 mm × 0.2 mm, hence $p_{0}$ has mm as unit. In the experimental configuration used θ is $∼1.8^{\circ }$, and therefore small angle approximation can be used to simplify strain calculation. Note that in this paper $p_{0}$ and $d_{0}$ were both set by placing the strain of the central position of the line scan to zero, since the middle point on the sample is likely to be close to strain-free state.

**Figure 4:**
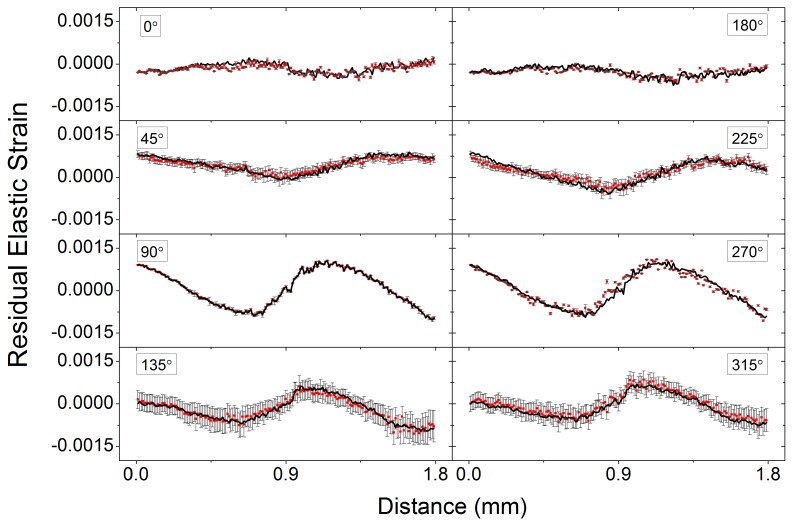
Comparison of residual elastic strains obtained from XRD-DIC (red dots with error bar) and ConFit method (solid black curve) at the azimuthal angles of $0^{\circ }$, $45^{\circ }$, $90^{\circ }$, $135^{\circ }$, $180^{\circ }$, $225^{\circ }$, $270^{\circ }$ and $315^{\circ }$ respectively.

The strain uncertainty for each azimuthal angle is calculated on the basis of the *sd* value of position evaluation using the approach described in the literature [39]. These values are used to generate the error bars shown in the strain profile plot in Figure 4 for different azimuthal angles.

## 4. Results and Discussion

The accuracy of XRD-DIC technique can be validated by comparison with the results of the conventional ConFit analysis. Figure 4 illustrates the comparison between strain results at the azimuthal angles $0^{\circ }$, $45^{\circ }$, $90^{\circ }$, $135^{\circ }$, $180^{\circ }$, $225^{\circ }$, $270^{\circ }$ and $315^{\circ }$ respectively. The horizontal axis is the distance across the sample. The conditional ConFit profiles are shown using black solid line, whilst the newly developed XRD-DIC results are shown as red dots with the associated error bars. Fit2D [45] and Matlab [46] were used for the conversion from 2D to 1D patterns and Gaussian fitting respectively in the ConFit method.

The XRD-DIC results clearly show good agreement with the results from the ConFit method, even when the absolute value of strain is relatively small, as is the case for the angles of $0^{\circ }$ and $180^{\circ }$ where strains fall below $5\times 10^{-4}$. It is interesting to observe that the error of strain determination varies for different angles. For example, the error bars are larger at azimuthal angles $45^{\circ }$, $135^{\circ }$, $225^{\circ }$ and $315^{\circ }$ than that of the other (‘square’) angles. This effect maybe associated with the nature of the DIC analysis process, but may also be affected by the presence of texture (preferred orientation) within the sample.

Whilst in the conventional lab XRD, a 1D line profile is obtained for the scattering vector located in the sample normal plane, in the case of high energy transmission XRD with 2D detector, the scattering vector directions form a conical surface with the half angle very close to $90^{\circ }$. This corresponds to scanning the scattering direction around $360^{\circ }$ in the plane of the sample, which includes the loading direction (horizontal), transverse direction (vertical), as well as all the intermediate directions, and they are all recorded in one 2D Debye-Scherrer ring pattern. In principle, the knowledge of three non-collinear strain values is enough to determine the in-plane principal strains and directions through fitting using Equation (5). However, fitting in this paper relies on data of 8 directions to improve the robustness of interpretation.

(5) $$\epsilon \left(\beta \right)=\frac{\epsilon_{1}-\epsilon_{2}}{2}\cos\left[2\left(\beta -\beta_{0}\right)\right]+\frac{\epsilon_{1}+\epsilon_{2}}{2}$$

β is the azimuthal angle and $\epsilon_{1}$ and $\epsilon_{2}$ are the unknown principal strains. In an ideal 3-point bending experiment, $0^{\circ }$ and $90^{\circ }$ in the current coordinate should be the principal directions. However, this is sometimes not exactly true in real cases because of reasons such as the misalignment of the sample during XRD experiment. To facilitate for this potential offset, the variable $\beta_{0}$ was introduced into Equation (5) as this offset angle and least squares fitting was used to determine an estimate.

**Figure 5:**
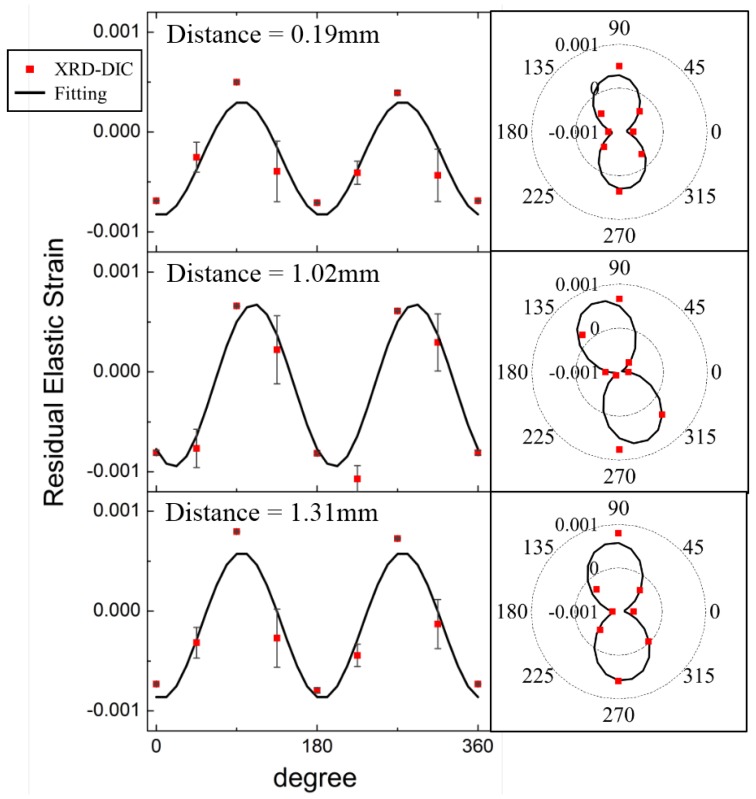
The azimuthal variation of residual elastic strains obtained using XRD-DIC from distance 0.19 mm, 1.02 mm and 1.31 mm as examples, showing the results of least squares fitting along with corresponding polar plots.

Figure 5 demonstrates the XRD-DIC strain estimates (red dots) and fitting result (black solid line) as a function of azimuthal angle for distance 0.19 mm, 1.02 mm and 1.31 mm (corresponding to images 19, 102 and 131) as examples, along with the corresponding radar plot. The values of $\beta_{0}$ for distance 0.19 mm, 1.02 mm and 1.31 mm are $5.6^{\circ }$, $19.2^{\circ }$ and $5.6^{\circ }$, respectively.

**Figure 6:**
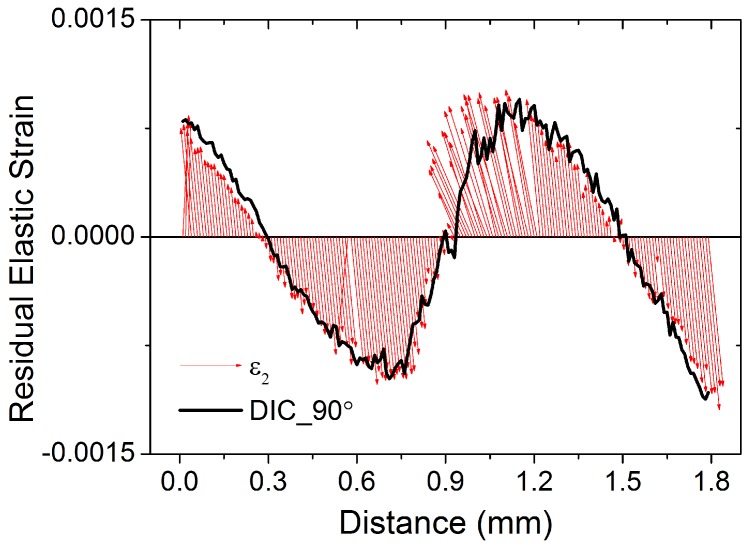
Quiver plot of in-plane principal strains across the central line in the bent bar plotted together with the residual elastic strain at $90^{\circ }$ calculated using XRD-DIC.

Figure 6 shows the ‘quiver plot’ of XRD-DIC residual strain results, as well as the results for the principal strains and directions. The black curve indicates the residual elastic strain values in the $90^{\circ }$ azimuthal direction calculated by XRD-DIC. The two principal strains are represented by the blue (ϵ1) and the red (ϵ2) arrows, whose length and direction indicates the magnitude and direction of the principal strains, respectively.

## 5. Conclusions and Outlook

In this paper a new approach for the analysis of 2D XRD data is proposed, developed and implemented, which we refer to as XRD-DIC. It offers a convenient and robust way of analysing XRD data for strain evaluation. The advantages of XRD-DIC stem from the fact that the conversion of 2D data set to the equivalent 1D intensity-2θ profile and fitting for peak position determination are no longer necessary. Instead, 2D patterns are analysed directly to monitor the radial shift in the Debye-Scherrer diffraction ring position by correlation image tracking. As a result, errors are minimised. Meanwhile, ‘caking’ operation for obtaining strain values at different azimuthal angles that is required in the conventional conversion and fitting procedure is obviated when using XRD-DIC. Lattice strains can be analysed using this approach for at as many angles as needed in one analysis, preparing the data input for full in-plane strain evaluation. The strain results attained from XRD-DIC and ConFit approaches show good agreement, even when low magnitude ($5\times 10^{-4}$) strains were examined, as illustrated in Figure 4. The principal directions of strains in a 3-point-bent bar were aligned with the axial and transverse directions with a small angular difference. The full in-plane strain state at each point of the scan line was obtained using the complete strain data extracted by fitting the results to the azimuthal strain variation according to Equation (5) that corresponds to Mohr’s circle construction.

It is concluded that XRD-DIC offers a reliable and accurate approach to the analysis of XRD data for the purpose of strain evaluation. The approach is beneficial in several aspects, namely, in that it (a) simplifies the analysis process by omitting the ‘caking’ conversion between 2D and equivalent 1D data, saving effort and minimising possible error sources associated with ‘caking’, and (b) allows evaluating the full in-plane strain state efficiently.

In terms of the outlook for the future use of the XRD-DIC approach proposed herein, it is worthwhile considering the effect of grain statistics within the sampling volume on the quality of 2D diffraction pattern and its interpretation. Grainy diffraction patterns arise when the number of grains that satisfy Bragg scattering condition within the sampling volume is low. This is known to cause problems for the evaluation of average lattice strain using the ConFit approach as grainy Debye-Scherrer rings give rise to poor quality of peaks on the 1D plot, leading to large scatter in the Gaussian peak fitting results. An interesting potential scope for future XRD-DIC analysis is to interpret diffraction patterns consisting of grainy rings in order to extract strain information from them. This approach may be combined with grain tracking, and also with sample tilting in order to obtain full 3D information in the reciprocal space.

## References

[1] Mishurova T., Cabeza S., Artzt K., Haubrich J., Klaus M., Genzel C., Requena G., Bruno G. (2017). An assessment of subsurface residual stress analysis in SLM Ti-6Al-4V. Materials.

[2] Ma Y., Song W., Bleck W. (2017). Investigation of the Microstructure Evolution in a Fe-17Mn-1.5 Al-0.3 C Steel via In Situ Synchrotron X-ray Diffraction during a Tensile Test. Materials.

[3] Yi S.B., Davies C., Brokmeier H.G., Bolmaro R., Kainer K., Homeyer J. (2006). Deformation and texture evolution in AZ31 magnesium alloy during uniaxial loading. Acta Mater..

[4] Bohr J., Feidenhans R., Nielsen M., Toney M., Johnson R., Robinson I. (1985). Model-independent structure determination of the InSb (111) 2 × 2 surface with use of synchrotron X-ray diffraction. Phys. Rev. Lett..

[5] Guss J.M., Merritt E.A., Phizackerley R.P., Hedman B., Murata M., Hodgson K.O., Freeman H.C. (1988). Phase determination by multiple-wavelength X-ray diffraction: Crystal structure of a basic “blue” copper protein from cucumbers. Science.

[6] Yang X.Q., McBreen J., Yoon W.S., Grey C.P. (2002). Crystal structure changes of LiMn 0.5 Ni 0.5 O 2 cathode materials during charge and discharge studied by synchrotron based in situ XRD. Electrochem. Commun..

[7] Shin H.C., Park S.B., Jang H., Chung K.Y., Cho W.I., Kim C.S., Cho B.W. (2008). Rate performance and structural change of Cr-doped LiFePO 4/C during cycling. Electrochim. Acta.

[8] Langford J.I., Louer D. (1996). Powder diffraction. Rep. Prog. Phys..

[9] Daum R., Chu Y., Motta A. (2009). Identification and quantification of hydride phases in Zircaloy-4 cladding using synchrotron X-ray diffraction. J. Nucl. Mater..

[10] Almer J., Lienert U., Peng R.L., Schlauer C., Odén M. (2003). Strain and texture analysis of coatings using high-energy X-rays. J. Appl. Phys..

[11] Xie M. (2014). X-ray and Neutron Diffraction analysis and Fem Modelling of Stress and Texture Evolution in cubic Polycrystals. Ph.D. Thesis.

[12] Shi X., Ghose S., Dooryhee E. (2013). Performance calculations of the X-ray powder diffraction beamline at NSLS-II. J. Synchrotron Radiat..

[13] Wanner A., Dunand D.C. (2000). Synchrotron X-ray study of bulk lattice strains in externally loaded Cu-Mo composites. Metall. Mater. Trans. A.

[14] Poshadel A., Dawson P., Johnson G. (2012). Assessment of deviatoric lattice strain uncertainty for polychromatic X-ray microdiffraction experiments. J. Synchrotron Radiat..

[15] Hutchinson J. (1968). Plastic stress and strain fields at a crack tip. J. Mech. Phys. Solids.

[16] Hill R. (1998). The Mathematical Theory of Plasticity.

[17] Macherauch E. (1986). Introduction to Residual Stress. Advances in Surface Treatments: Technology-Application-Effect.

[18] Zhang S.Y. (2008). High Energy White Beam X-ray Diffraction Studies of Strains in Engineering Materials and Components. Ph. D. Thesis.

[19] Rendler N., Vigness I. (1966). Hole-drilling strain-gage method of measuring residual stresses. Exp. Mech..

[20] Sebastiani M., Eberl C., Bemporad E., Pharr G.M. (2011). Depth-resolved residual stress analysis of thin coatings by a new FIB–DIC method. Mater. Sci. Eng. A.

[21] Korsunsky A.M., Sebastiani M., Bemporad E. (2010). Residual stress evaluation at the micrometer scale: Analysis of thin coatings by FIB milling and digital image correlation. Surf. Coat. Technol..

[22] Cheng W., Finnie I. (2007). Residual Stress Measurement and the Slitting Method.

[23] Treuting R., Read W. (1951). A mechanical determination of biaxial residual stress in sheet materials. J. Appl. Phys..

[24] Window A.L., Holister G.S. (1982). Strain Gauge Technology.

[25] Cullity B.D. (1956). Elements of X-ray Diffraction.

[26] Korsunsky A.M., Wells K.E., Withers P.J. (1998). Mapping two-dimensional state of strain using synchroton X-ray diffraction. Scr. Mater..

[27] Comley A., Maddox B., Rudd R., Prisbrey S., Hawreliak J., Orlikowski D., Peterson S., Satcher J., Elsholz A., Park H.S. (2013). Strength of shock-loaded single-crystal tantalum [100] determined using in situ broadband X-ray Laue diffraction. Phys. Rev. Lett..

[28] Daniels J., Drakopoulos M. (2009). High-energy X-ray diffraction using the Pixium 4700 flat-panel detector. J. Synchrotron Radiat..

[29] He B.B. (2011). Two-Dimensional X-ray Diffraction.

[30] Petit J., Bornert M., Hofmann F., Robach O., Micha J., Ulrich O., Le Bourlot C., Faurie D., Korsunsky A., Castelnau O. (2012). Combining Laue microdiffraction and digital image correlation for improved measurements of the elastic strain field with micrometer spatial resolution. Procedia IUTAM.

[31] Zhang F., Castelnau O., Bornert M., Petit J., Marijon J., Plancher E. (2015). Determination of deviatoric elastic strain and lattice orientation by applying digital image correlation to Laue microdiffraction images: The enhanced Laue-DIC method. J. Appl. Crystallogr..

[32] Pan B., Qian K., Xie H., Asundi A. (2009). Two-dimensional digital image correlation for in-plane displacement and strain measurement: A review. Meas. Sci. Technol..

[33] Lunt A.J., Baimpas N., Salvati E., Dolbnya I.P., Sui T., Ying S., Zhang H., Kleppe A.K., Dluhoš J., Korsunsky A.M. (2015). A state-of-the-art review of micron-scale spatially resolved residual stress analysis by FIB-DIC ring-core milling and other techniques. J. Strain Anal. Eng. Design.

[34] Yates J., Zanganeh M., Tai Y. (2010). Quantifying crack tip displacement fields with DIC. Eng. Fract. Mech..

[35] Abanto-Bueno J., Lambros J. (2002). Investigation of crack growth in functionally graded materials using digital image correlation. Eng. Fract. Mech..

[36] McCormick N., Lord J. (2010). Digital image correlation. Mater. Today.

[37] Kujawińska M., Malesa M., Malowany K. (2013). Measuring structural displacements with digital image correlation. SPIE Newsroom.

[38] Senn M., Eberl C. Digital Image Correlation and Tracking, 2015. https://cn.mathworks.com/matlabcentral/fileexchange/50994-digital-image-correlation-and-tracking?requestedDomain=true.

[39] Lunt A.J., Korsunsky A.M. (2015). A review of micro-scale focused ion beam milling and digital image correlation analysis for residual stress evaluation and error estimation. Surf. Coat. Technol..

[40] Lunt A.J., Salvati E., Ma L., Dolbyna I.P., Neo T.K., Korsunsky A.M. (2016). Full in-plane strain tensor analysis using the microscale ring-core FIB milling and DIC approach. J. Mech. Phys. Solids.

[41] Fong K.S., Atsushi D., Jen T.M., Chua B.W. (2015). Effect of Deformation and Temperature Paths in Severe Plastic Deformation Using Groove Pressing on Microstructure, Texture, and Mechanical Properties of AZ31-O. J. Manuf. Sci. Eng..

[42] Liu W., Wu G., Zhai C., Ding W., Korsunsky A.M. (2013). Grain refinement and fatigue strengthening mechanisms in as-extruded Mg–6Zn–0.5 Zr and Mg–10Gd–3Y–0.5 Zr magnesium alloys by shot peening. Int. J. Plast..

[43] Estrin Y., Vinogradov A. (2013). Extreme grain refinement by severe plastic deformation: A wealth of challenging science. Acta Mater..

[44] Mathworks Digital Image Correlation and Tracking, 2017. https://uk.mathworks.com/help/images/index.html.

[45] Hammersley A., Svensson S., Hanfland M., Fitch A., Hausermann D. (1996). Two-dimensional detector software: From real detector to idealised image or two-theta scan. Int. J. High Press. Res..

[46] (2016). Matlab Version R2016a.

